# Saratoga Springs, New York

**Published:** 1891-07

**Authors:** 


### SARATOGA SPRINGS, NEW YORK.

The general opinion that living at Saratoga is expensive deters many from going there, and especially is this true whenever the American Dental Association has selected this locality for its place of meeting. Knowing this idea to be erroneous, at our request Dr. A. C. Rich, of Saratoga, prepared the list on another page. This covers most of the prominent houses. Subsequent personal inquiry enables us to give the terms per day and week. By writing in advance no difficulty will be experienced in securing comfortable quarters at moderate cost.

We desire to express our thanks to Dr. Rich for his efforts to make this list satisfactory to those needing the information.

